# Causal Analysis of Multidimensional Dietary Data to Assess Effects on All-Cause Mortality

**DOI:** 10.3390/nu18101629

**Published:** 2026-05-21

**Authors:** Yohannes Adama Melaku, Zumin Shi

**Affiliations:** 1 Flinders Health and Medical Research Institute, Flinders University, Bedford Park, Adelaide, SA 5042, Australia; 2 Department of Nutrition Sciences, College of Health Sciences, QU Health, Qatar University, Doha P.O. Box 2713, Qatar; zumin@qu.edu.qa

**Keywords:** diet, dietary pattern, multivariate generalized propensity score, causal inference

## Abstract

**Background**: Methods applied under explicit causal assumptions can provide estimates that support potential causal interpretations of the effects of dietary factors on health outcomes. However, the high dimensionality inherent in dietary data presents a challenge. **Objectives**: Using multivariate analysis methods under causal assumptions, we identified dietary patterns and estimated their associations with all-cause mortality, as well as the effects of a 100 g/day increase in individual components. **Methods**: Data from 12,635 individuals aged 20 years and above from the National Health and Nutrition Examination Survey (NHANES), United States, were analyzed. K-means clustering was first used to identify dietary patterns, and then their associations with mortality risk were estimated both with and without inverse probability weighting (IPW). Second, the multivariate generalized propensity score (mvGPS) method was employed to estimate the average effects of dietary components on all-cause mortality under causal assumptions. Third, mutually adjusted models (non-mvGPS) were utilized to determine the effects of each dietary component. Relative risks (RR) and 95% confidence intervals (CI) were computed using fully adjusted Poisson generalized linear models. **Results**: In a 15-year follow-up period, 400 (3.2%) deaths were recorded. ‘Healthy’, ‘unhealthy,’ and ‘mixed’ dietary patterns were identified. Variations in estimates of ‘healthy’ and ‘unhealthy’ patterns with mortality were observed in non-IPW (RR = 0.96; 95% CI: 0.67–1.13 and RR = 0.79; 0.56–1.11) and IPW models (RR = 0.75; 0.55–1.04 and RR = 0.92; 0.63–1.36, respectively) compared to the ‘mixed’ pattern. In the mvGPS model, added sugar (RR = 1.21; 1.06–1.36), processed meat (RR = 1.20; 0.96–1.48), and legumes (RR = 0.82; 0.73–0.90) showed the strongest effects. Only whole grains (RR = 0.68; 0.46–0.98) had an effect in the non-mvGPS model. **Conclusions**: Applying mvGPS to multidimensional dietary data may help improve covariate balance across measured confounders and support more interpretable analysis of correlated dietary components. However, findings from this observational study should be interpreted as estimates under explicit causal assumptions, rather than definitive causal effects. Future studies should apply and further evaluate these approaches in larger and more diverse populations.

## 1. Introduction

Diet is a major modifiable determinant of longevity [1], yet its effects are rarely attributable to single nutrients in isolation. For this reason, dietary pattern research—capturing the combined intake of foods and beverages as they are commonly consumed—has become central to nutritional epidemiology and guideline development. Large evidence syntheses consistently show that patterns rich in vegetables, fruits, legumes, whole grains, nuts, and unsaturated fats (and lower in red and processed meats, refined grains, and added sugars) are associated with lower all-cause mortality, whereas more “Westernized” patterns tend to relate to higher risk [1,2,3].

These associations are biologically plausible, as diet plays a fundamental role in maintaining physiological homeostasis, supporting metabolic function, and regulating pathways related to inflammation, oxidative stress, and cardiometabolic health [4,5]. In epidemiological research, “healthy” dietary patterns are typically characterized by higher intakes of whole, minimally processed foods, while “unhealthy” patterns are defined by greater consumption of ultra-processed foods, added sugars, refined grains, and saturated or trans fats [5,6,7,8]. These contrasting dietary profiles are therefore hypothesized to differentially influence disease risk and survival, with sustained adherence to unhealthy diets potentially shortening life expectancy through increased risk of chronic disease and premature mortality [9].

However, estimating dietary pattern effects is complicated by the high dimensionality and correlation structure of dietary data [10]. Data-driven approaches (e.g., cluster analysis such as K-means) are increasingly used because they summarize complex intake without imposing an a priori scoring system, and they have been used to identify distinct food-group clusters that can be related to mortality outcomes [11,12]. However, such approaches primarily describe patterns and may not adequately address confounding or disentangle the independent effects of correlated dietary components, limiting causal interpretation. Moreover, derived patterns may differ across populations and analytic decisions, and results can be sensitive to modelling choices [13,14].

In addition to overall patterns, policy translation and public messaging often require quantification of the effects of specific dietary components simultaneously. This is particularly important because dietary guidelines are typically framed around individual foods or nutrients rather than abstract patterns. Moreover, understanding the joint effects of multiple components can help to identify which elements of a dietary pattern are most influential for health outcomes. However, causal estimation becomes harder when multiple correlated dietary components are considered jointly [15,16], as the dietary components may act antagonistically, independently, or synergistically. The generalized propensity score (GPS) extends propensity score to continuous exposures [17], enabling the estimation of exposure–response relationships. More recent research extends this framework to multiple continuous exposures via multivariate GPS (mvGPS), offering a framework and tools to balance confounding across several exposure components simultaneously [18,19,20].

Despite these advances, to the best of our knowledge, no studies have applied multivariate causal approaches to high-dimensional dietary data in the context of mortality; therefore, it remains unclear whether such methods provide more interpretable and robust estimates compared with conventional approaches. The present study applies clustering to derive dietary patterns and compares conventional and marginal estimates for all-cause mortality, while also using mvGPS (and mutually adjusted non-mvGPS models) to estimate the effects of key dietary components under causal assumptions. Unlike most previous studies that rely on single-exposure or conventional regression approaches, this study jointly models multiple correlated dietary components within a multivariate causal framework, alongside pattern-based analyses, to better characterize their independent and combined associations with mortality. While advancing causal methodology is a key objective, this study also aims to provide substantive insights into how individual dietary components and overall dietary patterns relate to mortality risk, with direct relevance for nutritional epidemiology and public health guidance. Furthermore, beyond methodological comparison, the direction and magnitude of associations are examined in relation to existing nutritional evidence to assess the substantive relevance of the findings.

In this study, the primary estimands were defined as population-average, or marginal, effects. For the dietary pattern analysis, the estimand was the marginal effect of membership in each dietary pattern compared with the reference pattern, estimated with and without inverse probability weighting. For the dietary component analysis, the estimand was the marginal effect of a 100 g/day increase in each dietary component on all-cause mortality, while accounting for the joint distribution and correlation of multiple dietary components using mvGPS. In contrast, the non-IPW and non-mvGPS models were interpreted as conventional conditional associations, included to provide comparison with the causal modelling approaches.

## 2. Materials and Methods

### 2.1. Study Design and Population

**Figure 1:**
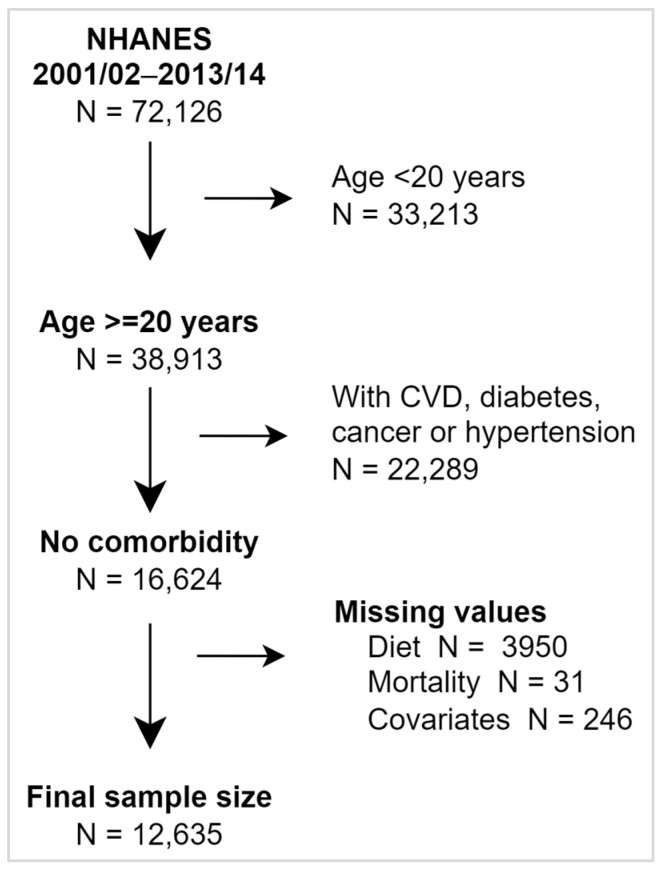
Sampling scheme (CVD—cardiovascular diseases; NHANES—National Health and Nutrition Examination Survey).

Data from the National Health and Nutrition Examination Survey (NHANES) were used. NHANES is a United States-based, repeated cross-sectional survey that has collected data through interviews, physical examinations, and laboratory assessments in continuous 2-year cycles since 1999/2000 [21]. Dietary data were collected only once in the 1999/2000 cycle, and mortality linkage was not available for the other cycles (post 2013/14). Therefore, our analysis was restricted to the 2001/02–2013/14 cycles [21,22]. Among 72,126 participants across the 2001/02–2013/14 cycles, 16,624 adults aged 20–78 years were free of cardiovascular disease, diabetes, cancer, and hypertension at baseline. After excluding participants with missing exposure, covariate, or outcome data, 12,635 participants were included in the final analysis (Figure 1). Participants were followed prospectively for mortality outcomes through linkage to the National Death Index, with a maximum follow-up of approximately 15 years. This allowed the analysis to be conducted within a prospective cohort framework.

### 2.2. Dietary Data

In each NHANES cycle, dietary intake was assessed using two non-consecutive 24 h dietary recalls, with the first conducted face-to-face and the second collected by telephone approximately 3–10 days later. Detailed procedures have been described previously [22]. Briefly, dietary data were collected using the U.S. Department of Agriculture (USDA) Automated Multiple-Pass Method over two non-consecutive days, with the first recall conducted in person and the second by telephone [22]. The recall captures all foods and beverages consumed in the preceding 24 h, including portion sizes, preparation methods, and nutrient composition. For the present analysis, the average of the two-day dietary recall was used. Nutrient intakes were estimated using the USDA Food and Nutrient Database for Dietary Studies. In addition, participant data were linked to the USDA Food Patterns Equivalents Database, which disaggregates foods and beverages into 37 USDA Food Patterns components [23].

Fruit, vegetables, legumes, whole grains, fish, added sugar, refined grains, meat, and processed meat were selected as they represent core protective and harmful dietary components consistently prioritized in global comparative risk assessments and national dietary guidelines [9]. They have also well-established links with all-cause mortality in large systematic reviews/meta-analyses [24,25]. In addition, these dietary exposures are readily available in NHANES dietary databases [22,23].

### 2.3. Mortality

The United States mortality registry was linked to NHANES participants using probabilistic record linkage methods developed by the National Center for Health Statistics, with mortality follow-up available through 2019 [21].

### 2.4. Covariates (Confounding Variables)

Sociodemographic variables included age (<50/≥50 years), sex (male/female), race/ethnicity (Mexican American, other Hispanic, non-Hispanic White, non-Hispanic Black, other race including multi-racial), education (less than high school, high school/GED, more than high school), marital status (married/living with partner, widowed, divorced, separated, never married), and family poverty–income ratio (FPIR) derived from U.S. Department of Health and Human Services Federal Poverty Guidelines [26]. Behavioral factors included smoking status (never, former, current; defined using the >100 cigarettes lifetime criterion and current smoking) and physical activity level assessed using the Global Physical Activity Questionnaire, expressed as metabolic equivalent task minutes per week (insufficient < 600, moderate 600–<1200, sufficient ≥ 1200) [27,28], and alcohol intake (moderate/no: ≤1 drink/day for women or ≤2 for men; high: >1 for women or >2 for men). Sex-specific alcohol consumption thresholds were applied in accordance with established guidelines [23], as women generally experience higher blood alcohol concentrations than men for a given intake due to differences in body composition and alcohol metabolism. BMI was calculated from measured height and weight (obesity defined as BMI ≥ 30 kg/m^2^). Depression severity was assessed using the Patient Health Questionnaire-9 (none, mild, moderate, severe) [29].

### 2.5. Statistical Analysis

**Descriptive analysis**: Participant characteristics were summarized overall and by dietary cluster. Categorical variables were presented as proportion. Continuous variables were summarized as mean (SD) when approximately symmetrically distributed and as median when skewed. NHANES uses a complex, multistage sampling design. Survey design features (strata and PSUs) and sampling weights were not incorporated into the descriptive estimates because our primary analytic approach (mvGPS) does not readily accommodate the full complex survey design. Therefore, descriptive results should be interpreted as sample characteristics rather than nationally representative estimates.

**K-means**: Dietary patterns were empirically derived using k-means cluster analysis [30] based on intakes (g/day) of nine food groups: fruit, vegetables, legumes/nuts/soy, whole grains, processed meat, fish, added sugar, refined grains, and meat. Prior to clustering, pairwise correlations among food groups were examined using Spearman’s correlation, and all food-group variables were standardized (z-scores) to place them on a common scale and prevent variables with larger variances from dominating the clustering solution. The optimal number of clusters was evaluated by inspecting the within-cluster sum of squares (WSS) “elbow” criterion across candidate solutions, and by visual assessment of cluster separation. K-means models were fitted for k = 2 to 5 clusters using multiple random starts (nstart = 20) and a fixed random seed to ensure reproducibility. A three-cluster solution was selected as it provided a parsimonious and interpretable classification. Participants were assigned to clusters based on the nearest cluster centroid. To characterize the resulting dietary patterns, food-group intakes within each cluster were summarized as mean standardized values (z-scores) to facilitate comparison across components. The clusters were characterized descriptively using standardized mean intakes (z-scores) of each dietary component. As cluster analysis is an unsupervised method, no formal statistical tests were used to compare clusters. Instead, interpretation was based on the relative magnitude and direction of z-scores across dietary components to ensure meaningful and interpretable dietary profiles. These steps align with recommended practices to enhance stability, interpretability, and reproducibility in k-means clustering applications in nutritional epidemiology [14,30].

**Inverse probably treatment weighting (IPTW)**: To estimate the marginal effect of dietary pattern membership (k-means clusters) on all-cause mortality, we applied IPTW [31]. Propensity-score weights for the three-level exposure were estimated using the Covariate Balancing Propensity Score approach implemented in the *WeightIt* package, with stabilized weights and the combined NHANES dietary sampling weights incorporated as sampling weights. The propensity score model included race/ethnicity, sex, marital status, education, family poverty–income ratio, physical activity, smoking status, alcohol intake, depression, health insurance status, BMI, total energy intake (kcal/day), follow-up time, and an interaction between follow-up time and age. For comparison, a survey-weighted Poisson regression model with conventional covariate adjustment (non-IPTW) was fitted to examine the association between dietary cluster membership and mortality, adjusting for the same covariates listed above.

**Non-mvGPS (conventional analysis)**: Associations between dietary exposures and mortality were estimated using survey-weighted Poisson generalized linear models with a log link to obtain relative risks (RRs) and 95% confidence intervals (CIs). Dietary exposures (fruit, vegetables, legumes/nuts/soy, whole grains, fish, meat, refined grains, added sugar, and processed meat) were modelled per 100 g/day increment (e.g., intake/100). A 100 g/day increment was selected to provide a common and interpretable scale across dietary components and to facilitate comparison of effect estimates within the multivariable and mvGPS frameworks. Using the same scale makes interpretation more straightforward because each estimate represents the relative risk associated with the same absolute increase in intake, rather than different units across food groups. However, we acknowledge that this increment may not have equivalent practical or biological meaning across all foods.

Models were adjusted for race/ethnicity, sex, marital status, education, FPIR, physical activity, smoking, alcohol intake, depression, health insurance status, BMI, and total energy intake (kcal/day). To account for differential follow-up, models additionally included follow-up time and an interaction between follow-up time and age. Each food group was examined in separate adjusted models, and a mutually adjusted model including all food groups simultaneously was also fitted.

**mvGPS**: The selected dietary components were jointly modelled using the mvGPS to estimate their effects on all-cause mortality simultaneously [18,20]. mvGPS extends the generalized propensity score framework to multiple continuous exposures by estimating stabilized IPTWs that balance measured confounders across the joint exposure distribution, supporting estimation under causal assumptions when measured confounding, positivity, consistency, and model specification assumptions are considered plausible [18,20]. Conceptually, this approach enables estimation of the effect of each dietary component while accounting for their interdependence within overall dietary patterns. Importantly, the use of mvGPS in this context is intended not only to improve covariate balance for measured confounders but also to enhance the interpretability of diet–mortality relationships by disentangling the joint effects of correlated dietary components. In our analysis, the mvGPS was estimated from the exposure matrix (D) conditional on baseline covariates (C). The analysis was restricted to the region of common support across exposure distributions (*common* = *TRUE*). To limit the influence of extreme weights, IPTWs were trimmed at the 99th percentile (*trim_w* = *TRUE*; *trim_quantile* = 0.99), a commonly used approach to improve stability. The mvGPS R package (version 1.2.2) was used. As the package does not support incorporation of complex survey design features, analyses were conducted without applying NHANES sampling weights (i.e., unweighted analyses). Therefore, mvGPS estimates should be interpreted as internally valid estimates within the analytic sample, conditional on measured confounding and causal assumptions, rather than as nationally representative estimates.

The primary aim of the mvGPS analysis was to estimate the effects of multiple correlated dietary components on all-cause mortality while minimizing confounding under explicate causal assumptions. The underlying hypothesis was that the mvGPS approach would achieve better covariate balance across dietary exposures compared with conventional models (non-IPW and IPW), thereby improving the validity of causal inference.

Covariate balance was assessed across models using standard diagnostics, including Euclidean distance, maximum pairwise correlation, and absolute mean differences. The variables included in the balance assessment comprised key sociodemographic, behavioral, and health-related covariates (race/ethnicity, sex, marital status, education, FPIR, physical activity, smoking, alcohol intake, depression, health insurance status, BMI, and total energy intake (kcal/day), as well as the dietary exposure variables.

All analyses were conducted using R version 4.4.2 (R Foundation for Statistical Computing, Vienna, Austria).

## 3. Results

### 3.1. Baseline Characteristics

Dietary patterns were derived using data-driven cluster analysis (K-means). Participants were classified into three mutually exclusive groups (healthy, mixed, and unhealthy) according to similarities in their overall dietary profiles. Among 12,635 participants, 6879 (54.4%) were classified as mixed, 2576 (20.4%) as healthy, and 3180 (25.2%) as unhealthy. The unhealthy cluster was predominantly male (2383; 74.9%), younger (median age 35 years), less educated (≤high school: 1654; 52.0%), and more likely to smoke (1009; 31.7%), with higher intakes of processed meat (31.3 g/day), refined grains (247 g/day), added sugar (144 g/day), and energy (2880 kcal/day). In contrast, the healthy cluster was older (median age 42 years), more educated (>high school: 1921; 74.6%), had lower smoking prevalence (253; 9.8%), and higher intakes of fruit (316 g/day), vegetables (189 g/day), legumes (50.7 g/day), and whole grains (45.2 g/day), with lower intakes of processed meat (2.76 g/day) and added sugar (55.9 g/day). All-cause mortality was lowest in the healthy cluster (69; 2.7%) and highest in the mixed cluster (245; 3.6%) (Table 1).

### 3.2. Dietary Patterns

**Figure 2:**
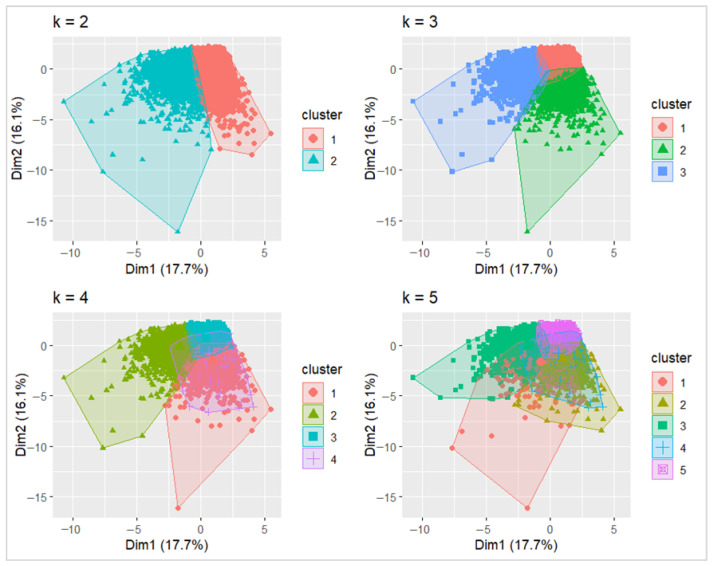
Standardized (z-score) dietary component profiles across clusters derived from k-means clustering (k = 3), based on food group intakes (g/day).

**Figure 3:**
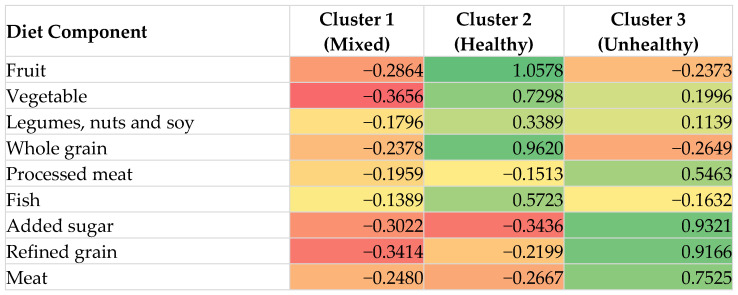
Standardized (z-score) dietary component profiles across clusters derived from k-means clustering (k = 3), based on food group intakes (g/day). Z-scores represent standardized dietary intakes (mean = 0, SD = 1) across the study population. Positive values (green) indicate higher-than-average intake, while negative values (red) indicate lower-than-average intake relative to the overall sample mean. Clusters were derived using k-means clustering based on standardized dietary variables.

The Spearman correlation matrix of dietary components is presented in Figure S1. Based on a combination of statistical criteria, cluster stability and interpretability, three clusters (dietary patterns: mixed, healthy and unhealthy) were selected in the K-means (Figure 2). Cluster characterization was based on relative differences in standardized mean intakes (z-scores). The healthy cluster was characterised by higher z-scores for fruits, vegetables, whole grains, legumes, and fish, and lower z-scores for processed meat, refined grains, added sugar, and meat. In contrast, the unhealthy cluster showed higher z-scores for processed meat, refined grains, added sugar, and meat, with lower or negative z-scores for most plant-based foods, while the mixed cluster generally showed modestly negative z-scores across most dietary components. This indicates generally lower-than-average consumption across both beneficial and less healthy food groups, rather than a clearly defined dietary pattern (Figure 3).

### 3.3. Associations of Diet and Mortality

**Figure 4:**
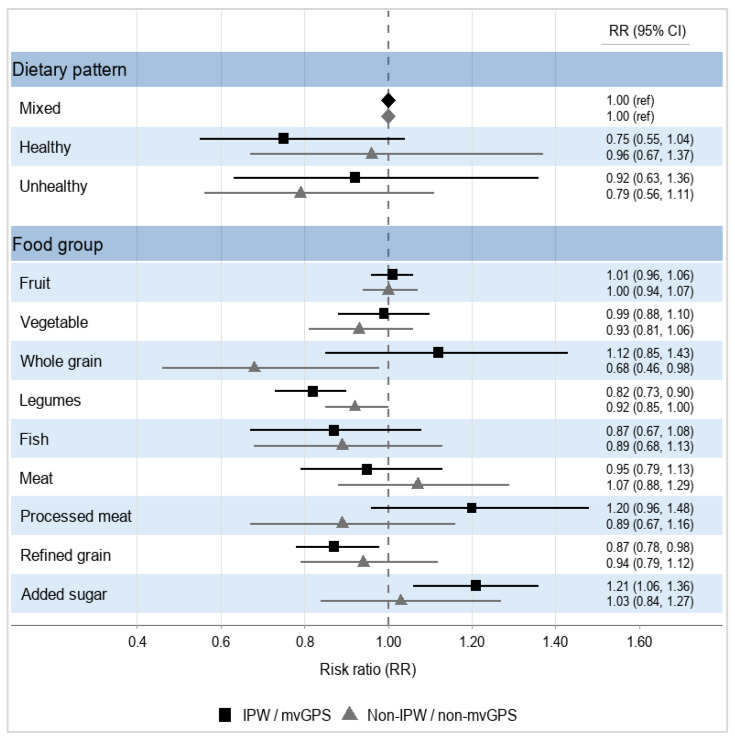
Associations of dietary patterns (IPW and non-IPW) and dietary components (mvGPS and non-GPS) with all-cause mortality, presented as relative risks (RR) and 95% confidence intervals (CI). IPW: inverse probability weighting; mvGPS: multivariable generalised propensity score. IPW (N = 12,635) estimates were weighted to account for confounding by baseline covariates, whereas non-IPW (N = 12,635) estimates are survey-weighted only. Relative risks (RRs) and 95% confidence intervals (CIs) are presented. The mixed dietary pattern was used as the reference category. Estimates for individual food groups represent the relative change in risk per 100 gm increase in intake (N = 5308 for mvGPS and 12,635 for non-mvGPS). Models were estimated using Poisson regression with a log link. IPW models were based on inverse probability weighting; mvGPS models used multivariate generalised propensity score weighting. All models were adjusted for sociodemographic and behavioural covariates.

In the IPW analysis, compared with the mixed cluster, the healthy cluster showed a lower risk of all-cause mortality during the follow-up period (RR = 0.75, 95% CI 0.55–1.04), whereas the unhealthy cluster showed no clear association (RR = 0.92, 95% CI 0.63–1.36). In the non-IPW model, the unhealthy dietary pattern tended to show a protective association with mortality during the follow-up period (RR = 0.79, 95% CI 0.56–1.11), although the estimate was imprecise (Figure 4). The protective association of the unhealthy dietary pattern in the non-IPW model should be interpreted cautiously, as it may reflect residual confounding or model instability rather than a causal protective effect.

In mvGPS models, higher legume intake was associated with lower mortality (RR = 0.82, 95% CI 0.73–0.90) over the follow-up period, while higher added sugar intake was associated with increased mortality (RR = 1.21, 95% CI 1.06–1.36). Corresponding non-mvGPS estimates were generally closer to the null (e.g., legumes: RR = 0.92, 95% CI 0.85–1.00; added sugar: RR = 1.03, 95% CI 0.84–1.27). Associations for grain intake differed by analytic approach: in mvGPS models, refined grain intake was inversely associated with mortality over the follow-up period (RR = 0.87, 95% CI 0.78–0.98), while whole grain intake showed no clear association (RR = 1.12, 95% CI 0.85–1.43). Meanwhile, in non-mvGPS models, whole grain intake was inversely associated with mortality (RR = 0.68, 95% CI 0.46–0.98) and refined grain intake was not. The inverse association for refined grains in the mvGPS model is more likely to reflect exposure correlation or unmodelled dietary substitution effects than a true protective effect. No association was observed between fruit and vegetable consumption and all-cause mortality during the follow-up period in either the mvGPS or non-mvGPS models (Figure 4).

### 3.4. Effectiveness in Confounder Balance

To assess the ability of different weighting approaches to reduce confounding, we evaluated covariate balance and dependence across dietary exposures using standard diagnostic measures (Table 2). The mvGPS approach achieved the best overall balance, with the lowest Euclidean distance (0.98), lower maximum correlation (0.41), and the smallest average correlation (0.04). These metrics indicate improved alignment of covariate distributions and reduced dependence between dietary exposures, supporting better approximation of the exchangeability assumption required for causal inference.

However, this improvement in balance was accompanied by a reduction in effective sample size (ESS = 5308), reflecting the increased variability of weights. In contrast, single-exposure propensity score models showed more modest improvements over the unweighted analysis, with Euclidean distances ranging from 1.21 to 1.31 and maximum correlations around 0.43–0.52, while largely preserving sample size (ESS = 9180 to 12,458). The unweighted model exhibited the greatest imbalance (Euclidean distance = 1.32), indicating substantial residual confounding.

Overall, the results presented in Table 2 demonstrate that the mvGPS approach provides superior confounder control across multiple correlated dietary exposures, albeit with some loss of precision, highlighting the trade-off between bias reduction and effective sample size.

## 4. Discussion

In our study of adults free of major chronic disease conditions at baseline, we identified three distinct dietary patterns using k-means clustering: healthy, mixed, and unhealthy. These patterns showed strong socio-demographic and behavioural gradients. When examining individual dietary components, mvGPS (designed to balance measured confounders across multiple correlated continuous dietary exposures) achieved the best overall balance, but with a substantial reduction in effective sample size. Within this framework, higher legume intake was associated with lower mortality, and higher added sugar and processed meat intake was associated with higher mortality over the follow-up period. These component-level findings were more consistent with prior evidence than several pattern-level estimates, suggesting that separating the “diet mixture” into interpretable components (while still balancing jointly) may help recover expected associations when dietary pattern classifications are heterogeneous. These findings are interpreted not only in terms of methodological performance but also in relation to established diet–mortality evidence, allowing us to evaluate whether advanced causal approaches yield more meaningful or coherent nutritional insights. Nevertheless, the protective association of the unhealthy dietary pattern in the non-IPW model and the inverse association for refined grains in the mvGPS model should be interpreted cautiously, as these unexpected findings are more likely to reflect residual confounding, model instability, exposure correlation, or unmodelled dietary substitution effects than causal protective effects. Furthermore, the observed associations relate baseline dietary intake to mortality outcomes during follow-up and do not capture changes in diet over time or the potential effects of sustained dietary changes.

### 4.1. Comparison with Previous Studies

The direction of association for the healthy pattern is consistent with large evidence syntheses showing that nutrient-dense dietary patterns (typically higher in plant foods and lower in processed foods and added sugars) are associated with lower all-cause mortality [2]. While our pattern estimates were imprecise, the overall pattern profile aligns well with what has been repeatedly observed across cohorts, including systematic reviews used to inform dietary guideline development [1,2,3]. Our component-level findings also agree with much of the literature. Higher legume intake has been associated with lower all-cause mortality in dose–response meta-analyses [32], supporting the inverse association observed under mvGPS. For added sugars, cohort evidence and meta-analyses indicate higher sugar intake is associated with higher all-cause and cardiovascular mortality [33], which is consistent with the positive association we observed over the follow-up period for added sugar under mvGPS. Similarly, higher processed meat intake has been linked to increased mortality and cardiometabolic risk in prospective studies and meta-analyses [34], aligning with the elevated mvGPS estimate in our analysis. However, an evidence synthesis of studies up to 2018 rated the certainty of evidence as low and found that associations with mortality and cardiometabolic outcomes were small [35], which may partly explain the attenuated associations observed in our study.

In contrast, the lack of a clear harmful association for the unhealthy dietary pattern differs from many studies where “Western” patterns are associated with increased mortality. One explanation is that cluster-derived patterns can be sensitive to analytic decisions and may capture broad behavioral profiles rather than a pure dietary gradient [14]. A second explanation is the limited statistical power for pattern-level comparisons (400 deaths in total) and within-cluster heterogeneity (e.g., the unhealthy cluster may include subgroups with different risk profiles despite similar mean intakes). A third explanation is residual confounding or measurement error, which can be especially problematic for diet (self-report) and for behaviors correlated with diet (smoking, alcohol, physical activity). For grains, prior evidence generally supports whole grains as protective for mortality and cardiometabolic outcomes [36]; however, we did not observe this expected inverse association in the mvGPS estimates from our analysis. The inconsistent findings across our methods—particularly the inverse association for whole and refined grains under mvGPS—should therefore be interpreted cautiously and likely reflect a combination of residual confounding, substitution effects, and sensitivity to weighting restrictions. Notably, the mvGPS-based estimates appeared to recover more consistent and biologically plausible associations for key dietary components, suggesting that methodological refinement may meaningfully improve substantive inference rather than serving purely technical purposes.

Despite consistent evidence from prospective cohorts and meta-analyses that higher fruit and vegetable consumption is associated with lower all-cause mortality [37], we did not observe a beneficial association for fruit or vegetables in either the mvGPS or the conventional (non-mvGPS) estimates over the follow-up period. This may reflect limited exposure contrast, because fruit and vegetable intakes may be generally low, leaving few participants at higher intakes where benefits are more detectable. In addition, measurement error (e.g., day-to-day variability and under-reporting in dietary assessment) can attenuate associations, particularly when the true intake range is narrow. It is also plausible that fruit and vegetables act mainly through dietary substitution (replacing more harmful foods) and broader dietary context; when models do not fully capture what foods are displaced, estimated “main effects” can appear null [38]. On the other hand, we did observe a higher contribution of fruit and vegetables within the healthy dietary pattern cluster, and this cluster showed an inverse association with all-cause mortality. This suggests that the protective signal may be better captured at the pattern level—reflecting correlated behaviors and food combinations—rather than by single food groups considered in isolation.

### 4.2. Implications

A key contribution of this work is methodological: it highlights how estimated diet–mortality associations can shift depending on whether the analysis targets a conventional conditional association or a marginal weighted estimand under causal assumptions. IPTW is widely used to improve covariate balance and estimate causal contrasts under exchangeability, positivity, consistency and correct weight-model specification, but it can be sensitive to extreme weights and the accompanying bias–variance trade-offs (including reduced effective sample size) [15]. We attempted to mitigate this using CBPS-based estimation for the multi-level pattern exposure, which directly targets balance while modelling treatment assignment [39].

For multi-component diets, the challenge is compounded by strong correlation. The generalized propensity score provides a framework for continuous exposures [17], and mvGPS extends this idea to multiple continuous exposures to balance confounders across the joint exposure distribution [20]. Our results illustrate both the promise and practical constraints of mvGPS: improved balance and more distinct exposure–outcome signals for legumes, added sugar and processed meat, but reduced effective sample size and potential sensitivity to overlap restrictions and trimming decisions.

From a public health standpoint, the component findings support familiar, actionable messages: promoting legumes and limiting added sugars and processed meat remain plausible priorities for chronic disease prevention and longevity, consistent with broader evidence and guideline directions [1,2,3,25,32]. However, the discordance between pattern-level and component-level findings suggests caution when interpreting “dietary clusters” as causal exposures: clusters may combine diet with correlated social and behavioral factors in ways that make a single causal interpretation difficult. This apparent inconclusiveness at the pattern level likely reflects the inherent heterogeneity and complexity of dietary behaviors, rather than a lack of analytical sensitivity, and highlights the importance of component-level approaches for more interpretable inferences.

### 4.3. Future Directions

Several steps could strengthen causal interpretation and reproducibility in future work. First, given the sensitivity of inverse probability weighting to limited overlap and extreme weights, future analyses should report results under alternative trimming thresholds and compare stabilized with unsterilized weights to characterize the bias–variance trade-off more fully [40]. Second, the divergent findings for fruit, vegetables, whole grains, and refined grains may reflect underlying dietary substitution patterns. Therefore, future studies using explicit isocaloric substitution models could provide more policy-relevant estimates. For instance, estimating the effect of replacing added sugars with legumes or whole grains, or substituting processed meat with legumes, could yield estimates that are more interpretable for guidelines and policy. This is because they reflect the practical dietary choices individuals and populations make when one food component is reduced and another is increased. Third, because under-reporting of energy intake in NHANES 24 h recalls is common and can vary by participant characteristics, sensitivity analyses that exclude likely under-reporters or incorporate reporting plausibility adjustments would help in the assessment of robustness. In addition, examining cardiovascular and cancer mortality separately may improve biological specificity compared with all-cause mortality alone. Finally, because data-driven dietary patterns can vary by population and analytic choices [14], replicating the clustering solution across NHANES cycles and validating it in external cohorts would strengthen confidence in the generalizability of the identified patterns and their associations.

### 4.4. Strengths and Limitations

Strengths include the use of a data-driven dietary pattern approach alongside component-based causal modelling, and transparent reporting of balance diagnostics showing that mvGPS improved confounder balance [20]. Key limitations include reliance on self-reported dietary recalls (subject to measurement error and misreporting), and limited events. The relatively small number of deaths limited precision, particularly for dietary pattern contrasts and for weighted analyses with reduced effective sample size. Dietary intake was assessed at baseline only, and changes in diet over the follow-up period could not be captured, which may lead to misclassification of long-term exposure and attenuate associations with mortality. Although key behavioral and sociodemographic factors were adjusted for, they were not modelled as primary exposures, and the multifactorial nature of mortality means residual confounding from non-dietary factors cannot be fully excluded. Additionally, mvGPS analyses could not incorporate the full NHANES complex survey design, so mvGPS results should be interpreted as internally valid estimates for the analytic sample rather than nationally representative effects. Furthermore, although this study emphasizes methodological advances, the observational nature of the data and measurement limitations inherent to dietary assessment constrain the strength of causal interpretation of diet–mortality relationships.

## 5. Conclusions

In this study, data-driven clustering identified distinct dietary patterns that reflected meaningful social and behavioral gradients, underscoring the value of dietary pattern approaches for characterizing real-world eating behaviors relevant to prevention. However, the main contribution of this study is methodological: it demonstrates the application of multivariate modelling to high-dimensional, correlated dietary exposures in relation to mortality under casual assumptions. While dietary pattern–mortality associations were broadly compatible with healthier eating being beneficial, these estimates were sensitive to analytic approach and should therefore be interpreted cautiously. In contrast, component-based mvGPS analyses provided clearer and more policy-relevant signals, with higher legume intake associated with lower mortality and higher added sugar and processed meat intake associated with higher mortality over 15 years of follow-up. No clear protective associations were observed for fruit or vegetable intake in either the mvGPS or conventional estimates, possibly reflecting limited exposure contrast and generally low intake in the cohort. However, these foods contributed more strongly to the healthy dietary pattern cluster that showed an inverse association with all-cause mortality, suggesting that their benefits may be better captured at the pattern level and within broader dietary substitution contexts. Together, these findings suggest that multivariate frameworks under explicit causal assumptions can strengthen the interpretation of complex diet–health relationships and may help identify dietary components most relevant for public health guidance. The findings also support prevention strategies that prioritize increasing minimally processed plant foods and reducing processed meat and added sugars.

## Figures and Tables

**Table 1:** Baseline characteristics of participants by dietary pattern (cluster membership).

	Cluster 1 (Mixed)	Cluster 2 (Healthy)	Cluster 3 (Unhealthy)	Total
Characteristics	(*N* = 6879)	(*N* = 2576)	(*N* = 3180)	(*N* = 12,635)
**Sex**				
Male	2467 (35.9%)	1306 (50.7%)	2383 (74.9%)	6156 (48.7%)
Female	4412 (64.1%)	1270 (49.3%)	797 (25.1%)	6479 (51.3%)
**Age (year)**				
Median [Min, Max]	38.0 [20.0, 85.0]	42.0 [20.0, 82.0]	35.0 [20.0, 85.0]	38.0 [20.0, 85.0]
**Education**				
Less Than High School	1266 (18.4%)	324 (12.6%)	764 (24.0%)	2354 (18.6%)
High School Diploma (including GED)	1497 (21.8%)	331 (12.8%)	890 (28.0%)	2718 (21.5%)
More Than High School	4116 (59.8%)	1921 (74.6%)	1526 (48.0%)	7563 (59.9%)
**Race**				
Mexican American	1042 (15.1%)	369 (14.3%)	698 (21.9%)	2109 (16.7%)
Other Hispanic	710 (10.3%)	253 (9.8%)	266 (8.4%)	1229 (9.7%)
Non-Hispanic White	2992 (43.5%)	1114 (43.2%)	1416 (44.5%)	5522 (43.7%)
Non-Hispanic Black	1363 (19.8%)	346 (13.4%)	575 (18.1%)	2284 (18.1%)
Other Race—Including Multi-Racial	772 (11.2%)	494 (19.2%)	225 (7.1%)	1491 (11.8%)
**Marital status**				
Married/living with partner	3998 (58.1%)	1715 (66.6%)	1999 (62.9%)	7712 (61.0%)
Widowed	194 (2.8%)	63 (2.4%)	34 (1.1%)	291 (2.3%)
Divorced	645 (9.4%)	220 (8.5%)	212 (6.7%)	1077 (8.5%)
Separated	220 (3.2%)	56 (2.2%)	95 (3.0%)	371 (2.9%)
Never married	1822 (26.5%)	522 (20.3%)	840 (26.4%)	3184 (25.2%)
**Income (FPIR)**				
Mean (SD)	2.59 (1.64)	3.11 (1.68)	2.34 (1.57)	2.63 (1.65)
**Health insurance**	1745 (25.4%)	516 (20.0%)	1127 (35.4%)	3388 (26.8%)
**Physical activity**				
Sedentary	2374 (34.5%)	653 (25.3%)	928 (29.2%)	3955 (31.3%)
Moderate	832 (12.1%)	314 (12.2%)	278 (8.7%)	1424 (11.3%)
Vigorous	3673 (53.4%)	1609 (62.5%)	1974 (62.1%)	7256 (57.4%)
**Smoking**				
Never smoked	4235 (61.6%)	1743 (67.7%)	1595 (50.2%)	7573 (59.9%)
Ex-smoker	1156 (16.8%)	580 (22.5%)	576 (18.1%)	2312 (18.3%)
Smoker	1488 (21.6%)	253 (9.8%)	1009 (31.7%)	2750 (21.8%)
**Body mass index**				
Mean (SD)	28.2 (6.56)	26.7 (5.23)	28.1 (6.36)	27.9 (6.29)
**Depression**				
None	5569 (81.0%)	2292 (89.0%)	2584 (81.3%)	10,445 (82.7%)
Mild	626 (9.1%)	160 (6.2%)	287 (9.0%)	1073 (8.5%)
Moderate	493 (7.2%)	95 (3.7%)	230 (7.2%)	818 (6.5%)
Severe	191 (2.8%)	29 (1.1%)	79 (2.5%)	299 (2.4%)
**Fruit, gm/day**				
Median [Min, Max]	77.6 [0, 654]	316 [0, 2910]	70.0 [0, 1380]	109 [0, 2910]
**Vegetable, gm/day**				
Median [Min, Max]	92.3 [0, 470]	189 [0, 2040]	141 [0, 760]	118 [0, 2040]
**Legumes, gm/day**				
Median [Min, Max]	10.3 [0, 1460]	50.7 [0, 3160]	17.6 [0, 3870]	17.2 [0, 3870]
**Whole grain, gm/day**				
Median [Min, Max]	7.80 [0, 140]	45.2 [0, 516]	4.54 [0, 182]	11.6 [0, 516]
**Processed meat, gm/day**				
Median [Min, Max]	7.37 [0, 200]	2.76 [0, 329]	31.3 [0, 475]	11.5 [0, 475]
**Fish, gm/day**				
Median [Min, Max]	0 [0, 267]	0 [0, 900]	0 [0, 407]	0 [0, 900]
**Added sugar, gm/day**				
Median [Min, Max]	61.6 [0, 320]	55.9 [0, 419]	144 [0, 733]	73.6 [0, 733]
**Meat, gm/day**				
Median [Min, Max]	21.8 [0, 269]	12.8 [0, 375]	77.8 [0, 663]	31.2 [0, 663]
**Refined grain, gm/day**				
Median [Min, Max]	129 [0, 420]	137 [0, 724]	247 [0, 1150]	153 [0, 1150]
**Energy intake, kcal**				
Median [Min, Max]	1680 [212, 4420]	2200 [409, 10,000]	2880 [1390, 9480]	2020 [212, 10,000]
**All-cause mortality**	245 (3.6%)	69 (2.7%)	86 (2.7%)	400 (3.2%)

FPIR—Family Poverty–Income Ratio; GED—General Educational Development; gm/day—grams per day; kcal—kilocalories; Max—maximum; Min—minimum; *N*—number of participants; SD—standard deviation. Continuous variables are presented as mean (SD) or median [min, max], and categorical variables as number (percentage). No statistical tests were performed, as this table is intended for descriptive purposes only.

**Table 2:** Covariate balance diagnostics for dietary exposures before and after weighting using multivariate Generalize propensity score (mvGPS) and single-exposure propensity score (PS) methods.

Method	Euclidean Distance	Maximum Correlation	Average Correlation	Effective Sample Size
mvGPS	0.98	0.41	0.04	5308
Refined grain (PS)	1.27	0.43	0.06	9180
Added sugar (PS)	1.21	0.49	0.05	9788
Processed meat (PS)	1.26	0.49	0.05	11,952
Meat (PS)	1.24	0.49	0.05	11,626
Legumes, nuts, and soy (PS)	1.30	0.50	0.06	12,227
Fish (PS)	1.31	0.50	0.06	12,458
Whole grain (PS)	1.28	0.51	0.05	12,038
Fruit (PS)	1.29	0.51	0.05	12,006
Vegetable (PS)	1.28	0.52	0.05	11,590
Unweighted	1.32	0.50	0.06	12,635

Euclidean distance, maximum correlation, and average correlation summarize the degree of imbalance between dietary exposures and covariates before and after weighting. mvGPS denotes multivariate generalized propensity score weighting, while PS refers to single-exposure propensity score weighting. Lower values indicate better covariate balance. Effective sample size reflects the impact of weighting on sample size.

## Data Availability

Data used in this study can be accessed at: https://wwwn.cdc.gov/nchs/nhanes/ (accessed on 12 September 2025).

## Supplementary Materials

The following supporting information can be downloaded at: https://www.mdpi.com/article/10.3390/nu18101629/s1, Figure S1: Spearman correlation matrix showing pairwise associations among dietary components.

## Abbreviations

The following abbreviations are used in this manuscript:

ATE	Average Treatment Effect
BMI	Body Mass Index
CBPS	Covariate Balancing Propensity Score
CI	Confidence Interval
CVD	Cardiovascular Disease
ESS	Effective Sample Size
FNDDS	Food and Nutrient Database for Dietary Studies
FPIR	Family Poverty–Income Ratio
GED	General Educational Development
GPS	Generalized Propensity Score
GPAQ	Global Physical Activity Questionnaire
HHS	(U.S.) Department of Health and Human Services
IPTW	Inverse Probability of Treatment Weighting
IPW	Inverse Probability Weighting
MET	Metabolic Equivalent Task
mvGPS	Multivariable Generalized Propensity Score
NHANES	National Health and Nutrition Examination Survey
PIR	Poverty–Income Ratio
PSU	Primary Sampling Unit
RR	Relative Risk
SD	Standard Deviation
USDA	(U.S.) Department of Agriculture
WSS	Within-Cluster Sum of Squares
