# Supplementary material for: Causal Analysis of Multidimensional Dietary Data to Assess Effects on All-Cause Mortality
Source: Nutrients. 2026 May 21;18(10):1629. doi: 10.3390/nu18101629 (PMC13210281; doi:10.3390/nu18101629)
Supplement: Supplementary file 1 [file nutrients-18-01629-s001.zip › nutrients-4122097-supplementary.pdf]

Causal Analysis of Multidimensional Dietary Data to Assess Effects on All-Cause Mortality

Yohannes Adama Melaku [1,*], and Zumin Shi [2]

[1]Flinders Health and Medical Research Institute, Flinders University, Bedford Park, Adelaide, SA 5042, Australia
[2]Department of Nutrition Sciences, College of Health Sciences, QU Health, Qatar University, Doha P.O. Box 2713, Qatar; zumin@qu.edu.qa
*Correspondence: yohannes.melaku@flinders.edu.au; Tel.: +61-8-7421-9746

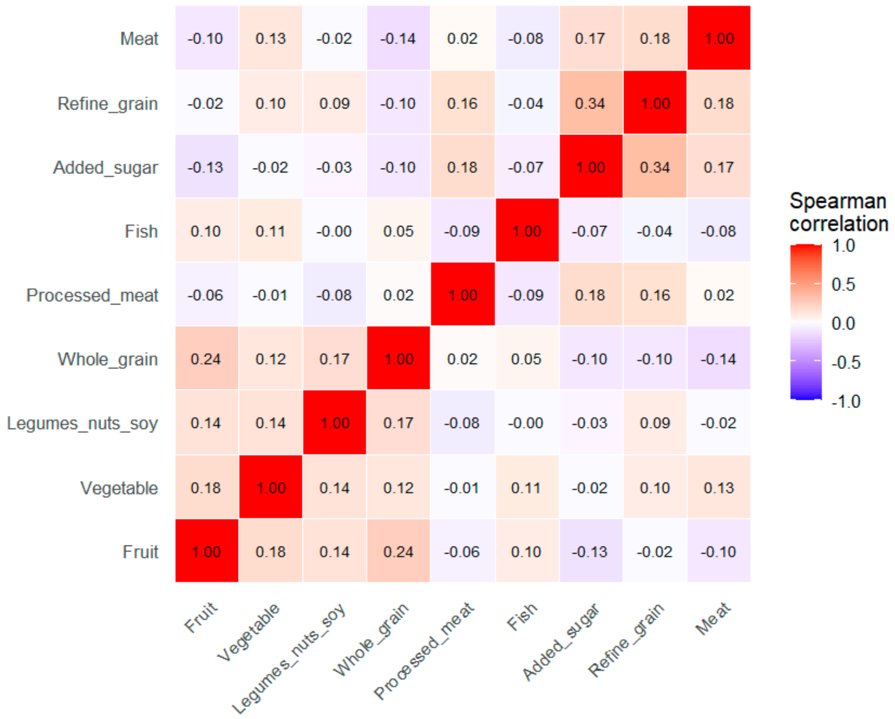

Figure S1| Spearman correlation matrix showing pairwise associations among dietary components
